# Motion characteristics of human roller skating

**DOI:** 10.1242/bio.037713

**Published:** 2019-02-20

**Authors:** Jiawei Chen, Kun Xu, Hanxin Ma, X. L. Ding

**Affiliations:** School of Mechanical Engineering and Automation, Beihang University, 37 Xueyuan Road, Beijing, 100191, China

**Keywords:** Human roller skating, Non-holonomic constraint, Skating inverted pendulum

## Abstract

In order to achieve a high speed of skating robot based on the leg structure – with passive wheels on even ground – the motion characteristics of human roller skating are studied in this paper. Utilizing a three dimensional motion capture system, the linear and turning gait are analyzed to express the motion characteristics of human roller skating. According to the observation and analysis, the normal linear gait can be divided into four phases and normal turning can be divided into three phases. The spin angle between the sagittal plane and the roller skate of the supporting leg is mainly generated by the external/internal rotation of the hip joint instead of the ankle, and the spin angular velocity of the body with one supporting leg is mainly generated by the roller angle of passive wheels. A new model we named the ‘skating inverted pendulum’ – based on the motion and angle features of the roller skating gait – is presented, which can be used to describe the characteristics of one supporting leg. The structure of roller skates and how they improve stability for roller skating, and the method of turning with a small radius are discussed.

## Abstract (precis)

**Summary:** The motion characteristics of human roller skating are studied using the theory of mechanism design. The skating inverted pendulum is used to describe roller skating with one supporting leg.

## INTRODUCTION

There are many studies (Baker, 2006) on human walking gaits from different perspectives. The kinematics analysis of human walking is a basis research (Mcginley et al., 2009; Nagahara et al. 2014) based on the theory of mechanism design. The Helen Hays model (Vaughan et al., 1999) is the most classic model used to describe the motion characteristics of human walking. Some research (Kadaba et al., 1990) focuses on the derivative form of the Helen Hays model, such as a the six-degrees-of-freedom gait analysis model (Żuk and Pezowicz, 2015) based on the International Society of Biomechanics’ recommendation on definitions of joint coordinate systems. The different conditions of human walking, such as slope walking (Lay et al., 2006) and stair walking (Yosibash et al., 2015), are an important research area. The research (Schache et al., 2007) discusses whether in fact the frame in which moments are expressed has a dominant effect upon transverse plane moments and thus provides a valid explanation for an observed inconsistency in the literature. Most studies on human walking observe the characteristics of gait and joint angle, and few investigate the mechanism of human walking. The spring-loaded inverted pendulum (SLIP) (Brown, 1986) is a famous simplified model used to describe animal running and is utilized to control biped and quadruped robots.

The wheel motion with high energy utilization on level ground is a special human creation, while the legged motion is the mechanism that animals use to walk. Much of the research into wheel motion focuses on special conditions such as the bicycle. The study by Kooijman et al. (2011) discusses the principle of bicycle self-stability that has not been elucidated in recent years.

The wheel-legged robot (Ding et al., 2017), which combines the structure of wheels and legs, has recently become a popular subject of research. The motion of passive wheels is a special locomotion which is different from the motion of active wheels. Human roller skating is an amusing special motion based on the leg structure with passive wheels to achieve a high speed on level ground instead of walking. The roller skating robot with high speed and adaptation becomes an interesting topic. The legged robot with passive wheels can be controlled in the same way as a common legged robot, and improvements in the efficiency of locomotion can be made with the addition of simple and light wheels. The ice skating humanoid robot (Iverach-Brereton et al., 2014) can propel itself on ice skates using a dynamically stable gait based on the inverted pendulum model, and the skating gait is better than a conventional walking gait. The leg-wheel hybrid vehicle with passive wheels, ‘Roller-Walker’ (Gen and Shigeo, 2012), can achieve a wheeled locomotion that improves efficiency by eight times in comparison to the crawl gait. The Roller-Walker is equipped with four passive wheels and has a static gait; however, the dynamic gait of roller skating is still difficult to achieve. So, the roller skating robot can learn from the motion characteristics of human roller skating. The research on human roller skating can promote a method to improve the velocity of a legged robot utilizing wheel motion.

Most research on human roller skating pays attention to energy and biological features. The experimental results (Stefanucci et al., 2008) suggest that explicit awareness of slant is influenced by the fear associated with a potentially dangerous action that could be performed on the hill. The research (Hettinga et al., 2011) is to ‘override’ self-paced performance by instructing athletes to execute a theoretically optimal pacing profile. Some research (de Koning et al., 2005) has evaluated these parameters during competitive imitations for the purpose of improving model predictions. Nobes et al. (2003) compares skating economy and oxygen uptake on-ice and on the skating treadmill. The research based on kinematic characteristics of roller skating is limited.

In this paper, the motion of roller skating is analyzed from the perspective of mechanisms utilizing a three dimensional (3D) motion capture system. According to the roller skating data, the standard skating gaits – linear and turning – are analyzed. Based on a new model derived from the Helen Hayes model, the supporting leg of roller skating can be simplified to a simple, dynamic model from examining the motion characteristics. The real structure of roller skates and the strategy of the turning gait with a small radius are discussed in detail.

## RESULTS

### The characteristics of the linear gait

According to the linear gait experiments of human roller skating, one linear gait cycle can be divided into four phases (Fig. S1).

### Right push phase (RPP)

The center of body is claimed as the critical parameter of the linear gait and moves from right leg to left leg through thrust generated by the right leg. The right roller skate has low relative motion with the ground when the thrust between the right skate and the ground is generated. Most of the vertical supporting force of the body is provided by the left leg. The right heel leaves the ground before the center of the body closes to the supporting skate.

### Right back phase (RBP)

The demarcation between RPP and RBP is the moment that the toe marker of the right skate leaves the ground. The body and left leg can be regarded as a rigid body without relative motion. The swing height of the right leg is as small as possible without the ground reaction force (GRF) in order to decrease energy consumption generated by wiggle. The center of the body follows the direction of the left passive wheels.

### Left push phase (LPP)

The demarcation between RBP and LPP is the moment that the heel of the right skate makes contact with the ground. The center of the body moves from the left leg to the right leg through thrust generated by the left leg. The left roller skate has no relative motion with the ground when the thrust between the right skate and ground is generated. Most of the vertical supporting force of the body is provided by the right leg. The heel of the left skate leaves the ground before the center of the body closes to the supporting skate.

### Left back phase (LBP)

The demarcation between LPP and LBP is the moment that the toe marker of the left foot leaves the ground. The body and right leg can be regarded as a rigid body without relative motion. The swing height of the left leg is as small as possible without GRF in order to decrease energy consumption generated by wiggle. The center of the body follows the direction of the right passive wheels. The demarcation between LBP and RPP is the moment that the heel of the left skate descends towards the ground.

**Fig. 1.**
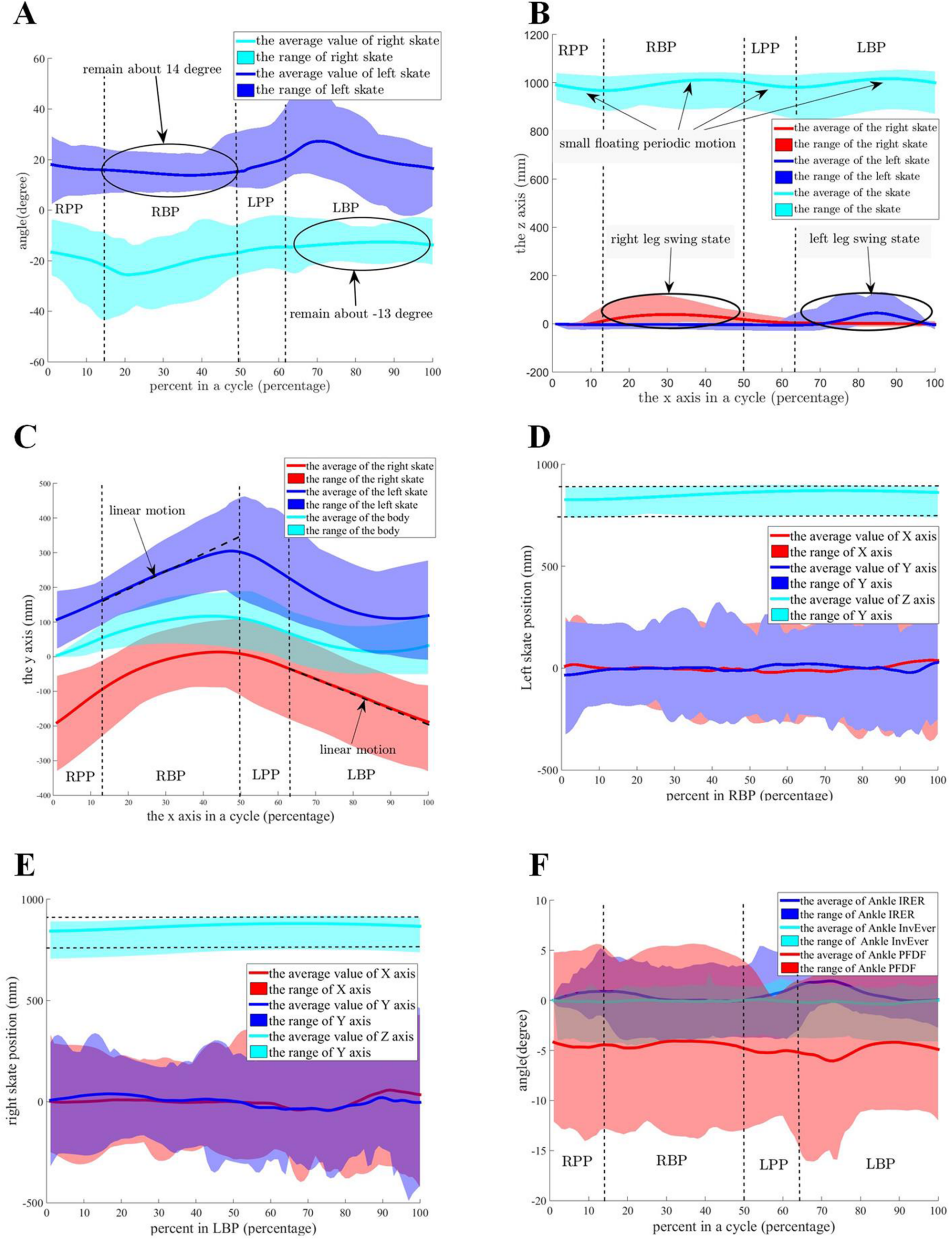
**The characteristics of the** **linear gait for human roller skating for all participants.** (A) The angle between the x axis of the body and the passive wheels in the cycle. (B) The z-x position of the roller skates and the center of the body in the ground coordination system. (C) The y-x position of the roller skates and the center of the body in the ground coordination system. (D) The relative position from the center of the body to the left skate in the skating coordination system in RBP. (E) The relative position from the center of the body to the right skate in the skating coordination system in LBP. (F) The angle of the ankle joint.

According to the data of the experimental results from all participants, the time of RPP, RBP, LPP and LBP are ∼10–15%, ∼35–40%, ∼10–15% and ∼35–40% of the whole cycle, respectively. The direction of the right and left passive wheels is shown in Fig. 1A in one linear gait cycle for all participants. In Fig. 1A, the angle between the x axis of body and the passive wheel of the supporting skate remains about 10–20°. The direction of the passive wheels of the supporting skate in LBP and RBP for five participants shown in Table 1 is the individual difference in the linear gait. The angle of the left and right skates have certain differences within a small range. In RBP and LBP, the body is controlled in order to be consistent with the supporting leg for a stable movement. The angle between the x axis of the body and the passive wheel of the swing leg, without obvious features, is dependent on the line motion for the body balance.

**Table 1.**
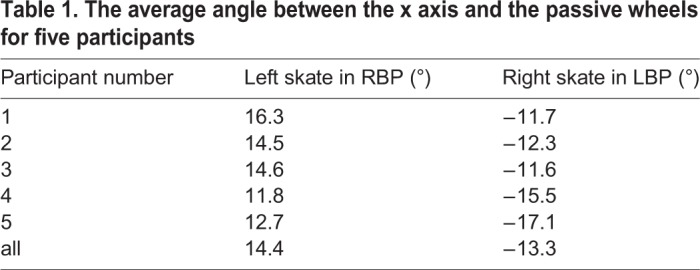
**The average angle between the x axis and the passive wheels for five participants**

The position of the roller skates and the center of body can be expressed as an ‘S’ curve in 3D space, as shown in Fig. 1B and C. In RBP and LBP, the value of the center of body along the y axis becomes bigger then smaller while the height of center of body becomes higher then lower. In the horizontal plane, roller skate motion is an apparent linear move along the direction of the supporting passive wheels in RBP and LBP. The skate height of the swing leg is ∼50 mm to reduce energy consumption.

The relative position from the center of body to the left roller skate in RBP and the relative position from the center of body to the right roller skate in LBP in the skating coordination system are shown in Fig. 1D and E. The relative position from the center of body to the supporting leg shows the same feature in the skating coordination system. In the skating coordination system, the value of the x axis and y axis has a large fluctuation. The value of the z axis shows a small fluctuation; it first rises and then falls, and remains ∼800 mm.

### The characteristics of the turning gait

According to the turning gait experiments of human roller skating, one turning gait cycle can be divided into three phases (Fig. S2).

### Enter turning state (ETS)

After the speed of the supporting passive wheels is high enough, the center of the body transfers to the left and the sagittal plane rotates to be parallel to the skating plane of the left leg by using hip rotation. The roll angle between the passive wheel of the left skate and the z axis of the body increases in order to gain a spin angular velocity of the body. The supporting force provided by the left leg gradually increases.

### Remain turning state (RTS)

This phase can be skipped while the expected spin angle of the body is small. When the roll angle between the passive wheel of the left skate and the z axis of the body remains a specific value, the body enters a state of equilibrium without any joint movement in the absence of friction loss. Most of the supporting force is provided by the left leg and the right leg is utilized to maintain balance. The angle between the right passive wheel and the body remains ∼15° in order to control the turning radius while the right foot is behind the center of the body along the *X*_*b*_ axis. Friction and initial speed determine the amount of time that the unchanged posture of the whole body can be maintained.

### Quit turning state (QTS)

When the aim direction of the turning gait is almost realized, the center of the body can return to the middle of the two legs. QTS can be regarded as the inverse process of ETS.

**Fig. 2.**
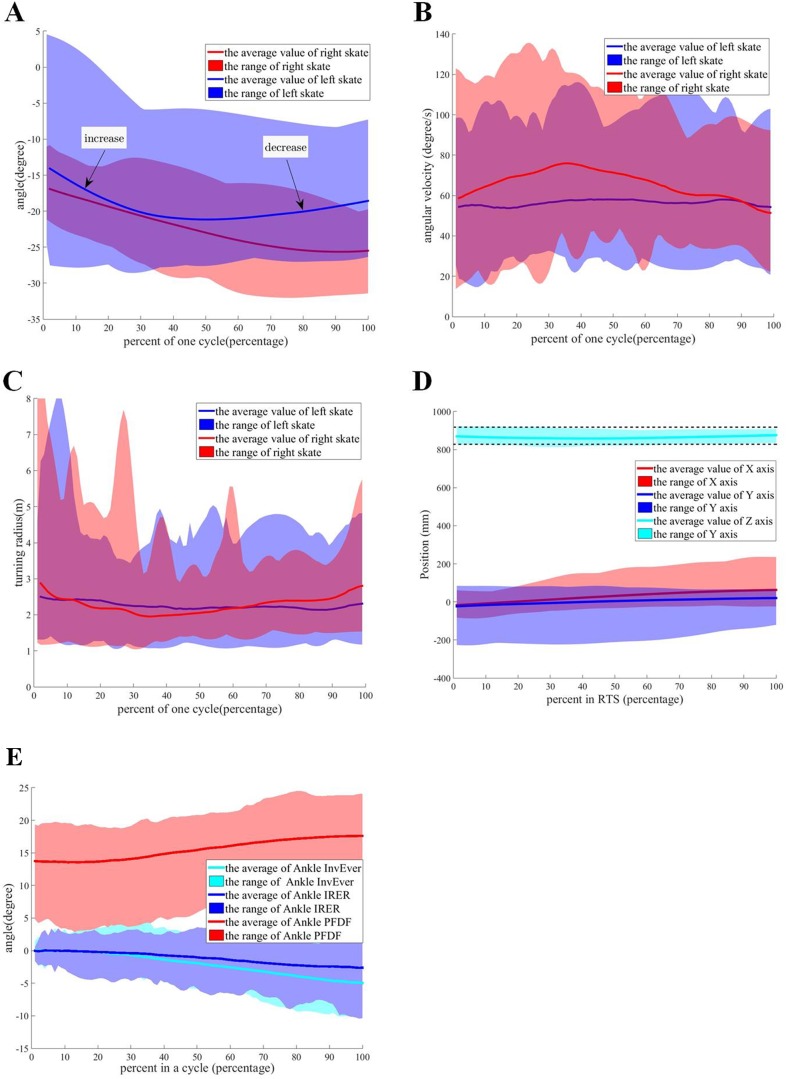
**The characteristics of the turning gait for human roller skating.** (A) The roll angle of passive wheels in the turning gait for all participants. (B) The spin angular velocity of the turning gait in a cycle for all participants. (C) The turning radius of the turning gait in a cycle for all participants. (D) The relative position from the center of body to left skate of RTS in the skating coordinate system. (E) The angle of ankle joint.

The roll angles of passive wheels in the turning gait are shown in Fig. 2A. The roll angle of the left skate increases when participants enter RTS and returns to the initial value when participants quit RTS. The roller angle of the left skate has a small change in RTS for a short time. The direction of the passive wheel of the right skate with tremendous float plays an important role in auxiliary balance and controlling the turning radius of the body. Because there is no relative motion between the center of the body and left skate in RTS, the spin angular speed of the left skate can be regarded as the spin angular speed of the body.

The spin angular velocity and turning radius of the passive wheel for all participants are expressed in Fig. 2B and C. In Fig. 2B, the spin angular velocity of the left skate remains ∼55°/s and the turning radius of the left skate is ∼2.3 m with a small floating in RTS. But the turning radius of the body varies a lot when the participant enters and quits the turning gait. A signalling disruption shown in the spin angular speed and turning radius of the right skate reveals the features of the non-holonomic constraint with sliding friction between the right skate and the ground. The angle between the x axis of the body and the passive wheel direction of the right skate is used to maintain the turning radius of the body using the feature of non-holonomic constraint. The kinematic characteristics of the right skate indicate the primary function of the right skate is to control the spin angular velocity of the body utilizing the lateral force of the passive wheel. This primary function is also confirmed by the fact that the angle between the left and right skate’s passive wheels remains at a constant value. The relative position between the left and right skates is another factor used to control the turning radius of the body. The turning radius and roll angle of the body are decided by the passive wheel speed of the left skate when the participant enters the turning gait. The turning gait is difficult to achieve when the initial speed of the passive wheel is too slow or too fast. The average spin angular velocity and turning radius of passive wheels in RTS for all participants is shown in Table 2. In order to achieve the same radius of circular movement, the spin angular velocity had to remain similar to each other.

**Table 2.**
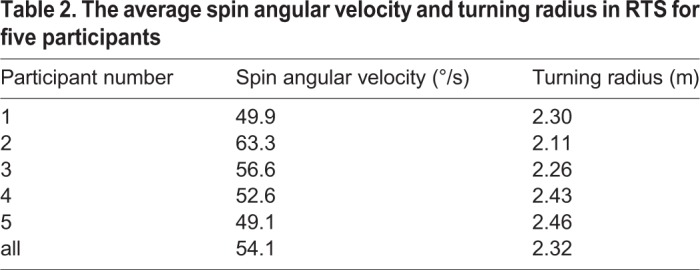
**The average spin angular velocity and turning radius in RTS for five participants**

The relative position from the center of the body to the left skate of RTS in the skating coordination system is shown in Fig. 2D. The relative position from the center of the body to the left skate of RTS remains unchanged for the stable turning radius. The value of *Z*_*S*_ axis shows a small fluctuation and remains ∼0.8 m. A small change shows the expected motion of the body while the participant wants to control the stability of the movement direction of the passive wheel along the *X*_*S*_ axis.

## DISCUSSION

### The joint feature of the roller skating gait

The motion characteristics of roller skating are decided by the bone structure of humans. The analysis of human bone structure based on the new roller skating model improves our understanding of the internal cause of the motion characteristics of roller skating. The roller skating model for expressing the motion of human roller skating is similar to the typical Helen Hayes model for describing the motion of human walking. Seven segments in the roller skating model consist of one pelvis, two thighs, two shanks and two roller skates. Three main joints, whose movements are expressed in the Materials and Methods section, are defined as the hip joint, knee joint and ankle joint, similar to the Helen Hayes model. The hip joint of the roller skating set connects the pelvis and a thigh. The knee joint connects a thigh and a shank. The ankle joint connects a shank and a roller skate.

The hip joint can be regarded as a spherical joint with three degrees of freedom (DOFs). The ankle and knee in the roller skating model are modified based on the features of roller skating. In the traditional Helen Hays model, the ankle joint can be regarded as a spherical joint with three DOFs. The three DOFs of the ankle joint are named as Ankle_PFDF, Ankle_InvEver and Ankle_IRER. Their variations in the linear and turning gait are shown in Figs 1F and 2E. In the linear gait, the Ankle_PFDF angle of the supporting leg remains constant with a large range for all passive wheels touching the ground. The angle of Ankle_InvEver and Ankle_IRER remains ∼0° with a small fluctuation when the leg is in a supporting state. In the turning gait, the Ankle_PFDF angle of the supporting leg changes following the height of the body with a large range for stability. The angle of Ankle_InvEver and Ankle_IRER shows a small fluctuation. According to the features of the angle joint for roller skating, the ankle joint can be regarded as a revolute joint named as Ankle_PFDF. In the roller skating model, Ankle_InvEver and Ankle_IRER of ankles that do not affect the motion characteristics of human roller skating can be ignored. In the traditional Helen Hays model, the knee joint can be regarded as a spherical joint with one DOF named as Knee_FE.

### The derivation of one supporting leg

**Fig. 3.**
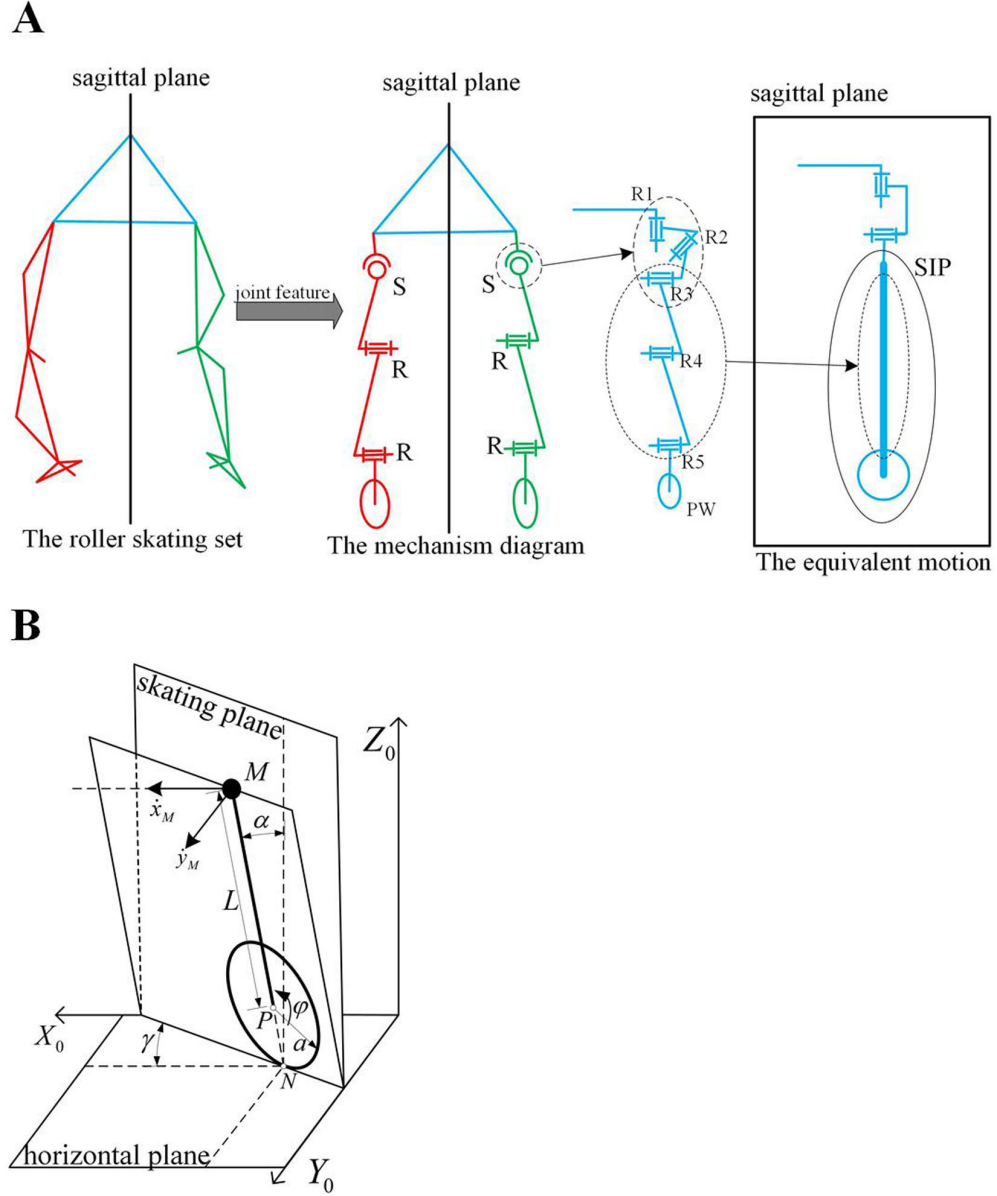
**The derivation and model of SIP.** (A) The derivation from real roller skating to SIP. (B) The model of SIP.

The simply wheel model is difficult to fit with the motion features of the center of the body with one supporting leg while roller skating. Based on the characteristics of body motion and all joints, a new model named the skating inverted pendulum (SIP) for one supporting leg was created to simplify roller skating characteristics. The derivation from real roller skating to the SIP model is shown in Fig. 3A. The hip, knee and ankle joints of the roller skating set can be regarded as a spherical, a revolute and a revolute joint based on the features of all joints, respectively. The passive wheel constraint (PW) of the supporting leg consists of one revolute and one typical non-holonomic constraint. The mechanism diagram of one supporting leg can be expressed as an S-R-R-PW serial mechanism from the traditional Helen Hays model (Fig. 3A). The axis of the passive wheel is perpendicular to the sagittal plane in the initial state. The axis of these two revolute joints are parallel to each other and are parallel to the axis of the passive wheel in the initial state. Based on the theory of mechanism design, one spherical joint can be broken down into three revolute joints named R1, R2 and R3. The axis of R1 is parallel to the direction of gravity. The axis of R2 is parallel to the direction of the passive wheel in the sagittal plane. The axis of R3 is parallel to R4. The combination of R3, R4 and R5 can be equivalent to an inverted pendulum that is perpendicular to the direction of the passive wheel in the sagittal. In the linear gait, R1 is utilized to adapt the angle between the sagittal plane and the direction of the passive wheel. Part of the spin force used to achieve the turning gait is generated by R1. R2 is used to generate the roller angle for the SIP model and the torque to keep the body balanced. SIP including R3, R4, R5 and PW is used to describe the motion characteristics of human roller skating. The SIP is different from the traditional plane locomotion on the vertical plane in 3D motion because the contact between the passive wheel and the ground is a non-holonomic constraint. The motion feature of SIP decides the characteristics of roller skating based on one supporting leg. When values of R1 and R2 are not equal to zero, the sagittal plane should change to the skating plane in showing the motion characteristics of the SIP model.

### The characteristics of the SIP model

The SIP model consists of an equivalent rigid body, an equivalent link and a virtual passive wheel, as shown in Fig. 3B.

In Fig. 3B, M, P and N are points at the center of the body, the center of the virtual passive wheel and the contact between the passive wheel, and the ground, respectively. The mass and moment of inertia of the equivalent body are *m*_*M*_ and 
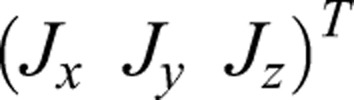
. The equivalent link, whose mass and moment is ignored, has a rigid connection with the rigid body. The virtual passive wheel, whose mass and moment is ignored, has a revolution joint with the equivalent link whose length is *L*. The radius and angle of passive wheel are *a* and *φ*. The orientation of the rigid body can be described as rotating *γ* about the z axis after rotating *α* about the x axis. Based on the feature of non-holonomic constraint, the kinematics of the rigid body can be expressed as:(6)
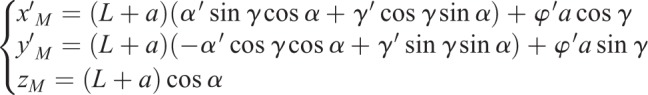
where 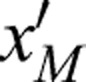, 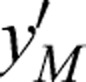 is the velocity of body along *X*_0_, *Y*_0_ axis and *z*_*M*_ is the height of the body.

According to the Lagrange equation, the dynamic of the rigid body about *α*, *γ*, φ can be expressed as:



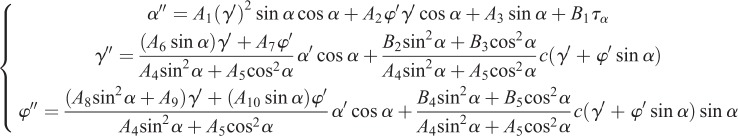


where *A*_*i*_(*i*=1, … …, 10), *B*_*i*_(*i*=1, ……, 5) is constant about 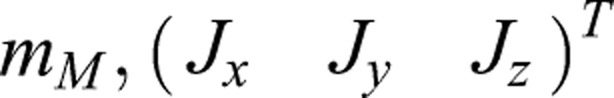, *τ*_*α*_ is the moment for the balance of the rigid body, *c* is the damping torque coefficient of the z axis generated from frictional resistance.

The dynamic model of SIP with an integral about the time is no analytic solution. We pay attention to the special stable state of SIP, the linear motion and circular motion.

### The linear motion

The boundary condition of the linear motion can be expressed as: 
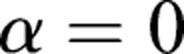
, 
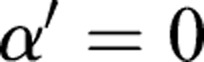
, 
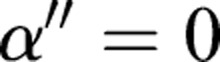
, 
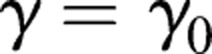
, 
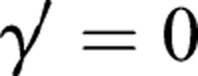
, 
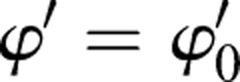
, satisfied the dynamic equation of SIP. *γ*_0_ is a constant and 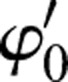 is a constant that is not zero. The motion of the rigid body that can achieve linear motion is described as:(8)
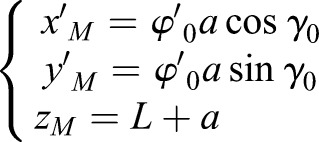
The special boundary condition of the linear motion is utilized to keep the linear gait of human roller skating with one supporting leg. The supporting leg of the linear gait shows the same features as the SIP model and moves along the direction of the passive wheels. In LBP and RBP, the center of the body is controlled by the line of supporting passive wheels. The axis of supporting passive wheels remained parallel to the horizontal plane.

### The circular motion

The boundary condition of the circular motion is similar to the linear motion, with a different initial value *γ*^′^≠0. The boundary condition of the circular motion can be expressed as: 
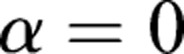
, 
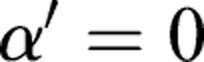
, 
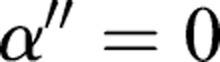
, 
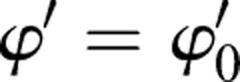
, 
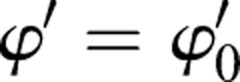
. 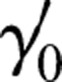 and 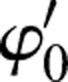 that is not zero is satisfied the condition that *α*^′′^≡0. The motion of the rigid body can achieve the circular motion described as:(9)
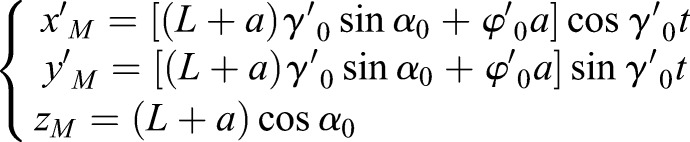
In the special circular motion, the trajectory of the rigid body is a circle in the horizontal plane. The turning radius of the center of the body in the boundary condition of the circular motion is:(10)
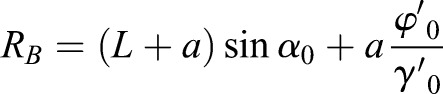
The turning radius of the circular motion is decided by 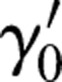 and 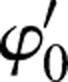, which is a free DOF without directly controlling. According to the dynamic of SIP from the linear motion to the circular motion, the spin velocity generated by roll velocity can be used to turn instead of using the direct control from the spin force. The spin angular velocity (*γ*′) of the center of the body can be generated by the hip joint with a huge torque. For improving the energy utilization in the turning gait, the special dynamic characteristic of SIP is used to generate the spin angular velocity. The spin angular speed adjusted by the roller angle from zero to 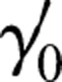 controls the turning radius with the passive wheel speed 
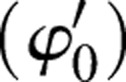
, as shown in Fig. S3.

### Strategy of roller skating

In this section, the method using SIP to enhance stability in the linear gait and efficiency in the turning gait is discussed.

**Fig. 4.**
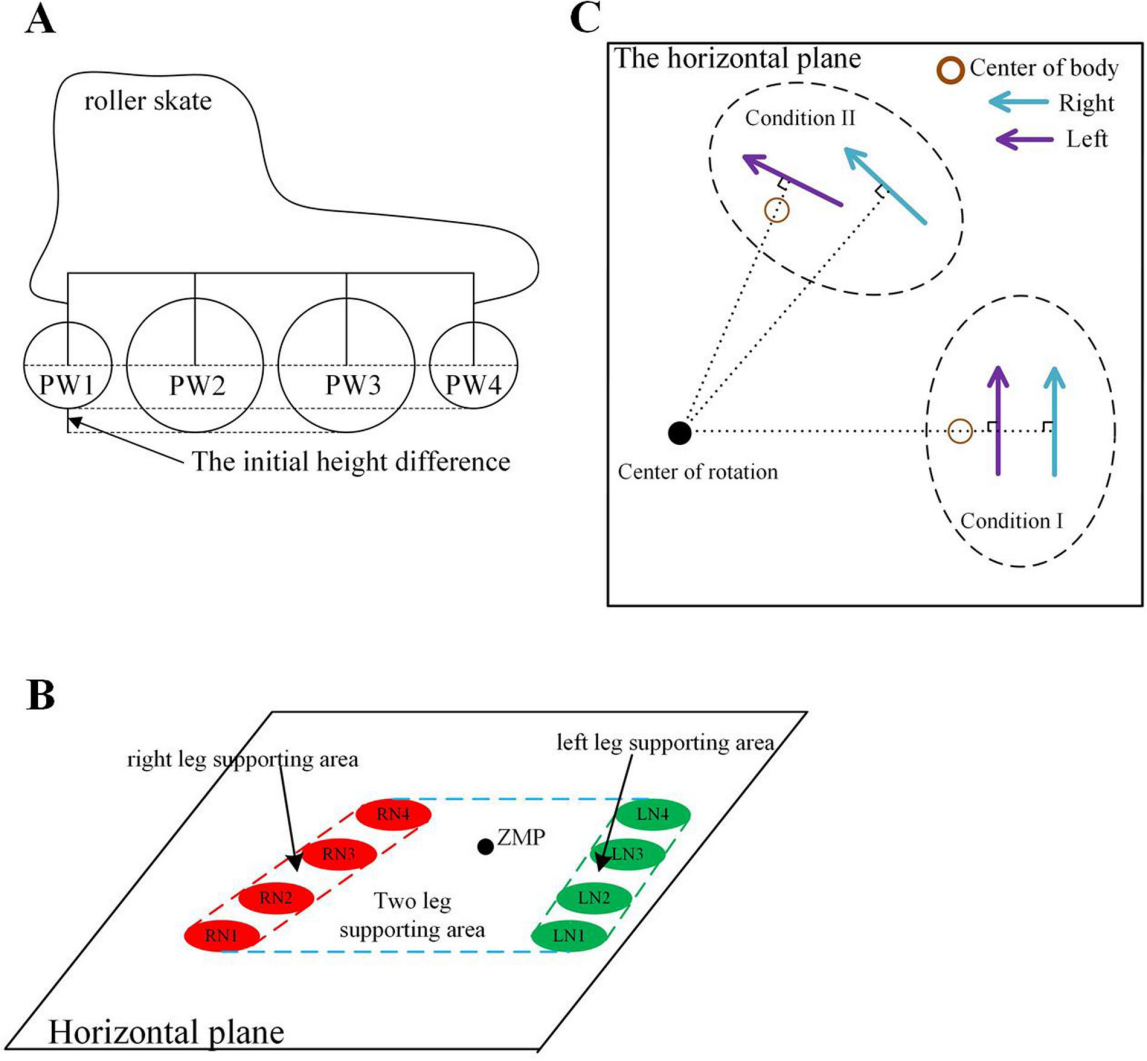
**The real structure of human roller skating.** (A) The real structure of a roller skate. (B) The supporting area of human roller skating. (C) The condition of cooperative work in RTS.

In order to analyze the stability of human roller skating, the zero moment point (ZMP) (Vukobratovic and Borovac 2004; Potkonjak 2012), used for analysis of human and robot motion, stability was selected to be the stability criterion. In contrast to traditional human walking, the contact area of the roller skate between one passive wheel and the ground is very small. So the structure with one passive wheel is different to maintain stability. In real roller skates, four passive wheels named PW1, PW2, PW3 and PW4 are collinear, as shown in Fig. 4A. The radius of PW2 and PW3 is greater than PW1 and PW4. The contact area between one roller skate and the ground, including four disconnected small areas, increased the supporting area. The supporting area of human roller skating based on ZMP is shown in Fig. 4B. The contact areas of the left skate PW1, PW2, PW3 and PW4 are LN1, LN2, LN3 and LN4. The contact areas of the right leg PW1, PW2, PW3 and PW4 are RN1, RN2, RN3 and RN4. The supporting area of human roller skating with two supporting skates, utilizing the structure of the roller skate, is close to the area used while walking. The human roller skating with two supporting skates has better stability, such as RPP and LPP. The supporting area with one supporting is close to a line, such as RBP and LBP. The stability of human roller skating with one supporting leg is very low. The movement of the supporting skate is important in controlling the ZMP for maintaining stability. The movement of R3, R4 and R5, equivalent to the planar motion, can adapt the equivalent inverted pendulum to be perpendicular to the direction of the passive wheel in the skating plane. The equivalent inverted pendulum is difficult to be perpendicular to the direction of the passive wheel while the supporting skate has one passive wheel. The stable area is proportional to the distance between the center points of PW1 and PW4. The stability of roller skating can be improved by the structure of the roller skate. So the mass pitching along the direction of the passive wheel is not considered in the SIP model. In the linear gait, the axis of the passive wheels of the supporting skate is parallel to the horizontal plane to achieve the linear motion based on SIP. The center of the body is controlled between the center position of PW1 and PW4 to maintain stability while roller skating.

Based on SIP, the rotating motion of roller skating can be generated by rolling passive wheels. However, the frictional resistance generated by the structure of four passive wheels is too large to easily achieve the turning gait with a small radius. The difference between four passive wheels and the cooperative work of two legs plays a significant part in achieving a turning gait with a small radius. The initial height difference can be utilized to decrease the frictional resistance while the touch point between the four passive wheels and the ground is not collinear. The touch point between the four passive wheels and the ground can be regarded as the point of circle arc in RTS for improving the effect of rotation. In RTS of the turning gait, the direction and position of passive wheels can be divided into two conditions shown in Fig. 4C. In condition I, the passive wheel axis of two legs is collinear. The turning gait with a small radius is difficult to obtain when the whole of the body can be regarded as a holonomic constraint of a rectilinear motion in condition I. Because the whole of the body can be regarded as a holonomic constraint of a rotary motion in condition II, it was selected to achieve a turning gait with a small radius by the participants in the experiment.

## CONCLUSION

Based on the motion characteristics of roller skating, linear gait can be divided into four phases and turning gait can be divided into three phases. The spin angle between the sagittal plane and the roller skate of the supporting leg is mainly generated by the external/internal rotation of the hip joint instead of the ankle. In RBP and LBP of the linear gait, the motion of the body and the supporting skate along the direction of the supporting passive wheels shows the same feature. In the turning gait, the spin angle of the body with one supporting leg is mainly generated by the roller angle of the passive wheels. The SIP model was built to describe the center of the body with one supporting leg in the linear gait and turning gait. Utilizing the structure of a roller skate with four passive wheels to improve stability, the SIP model ignores the DOF of rotating the y axis in the body coordination system. The method to achieve a turning gait with a small radius is to use the two non-holonomic constraints of two legs. The motion characteristics of human roller skating shares the same features as the SIP model during the special state. In this work, the roller skating robot used the SIP model and this method to improve its stability and efficiency.

## MATERIALS AND METHODS

### Subjects and materials

Five male participants (mean±s.d., 175±20 mm, 65±5 kg) used the same roller skates to achieve roller skating. Subjects provided written informed consent and study procedures were conducted in accordance with the Declaration of Helsinki. A typical roller skate with four passive wheels was selected to maintain the reliability of the experiment. The radius of first/last passive wheel was 38 mm and the radius of second/third passive wheel was 39 mm. The distance between the center points of two adjacent passive wheels was 80 mm.

**Fig. 5.**
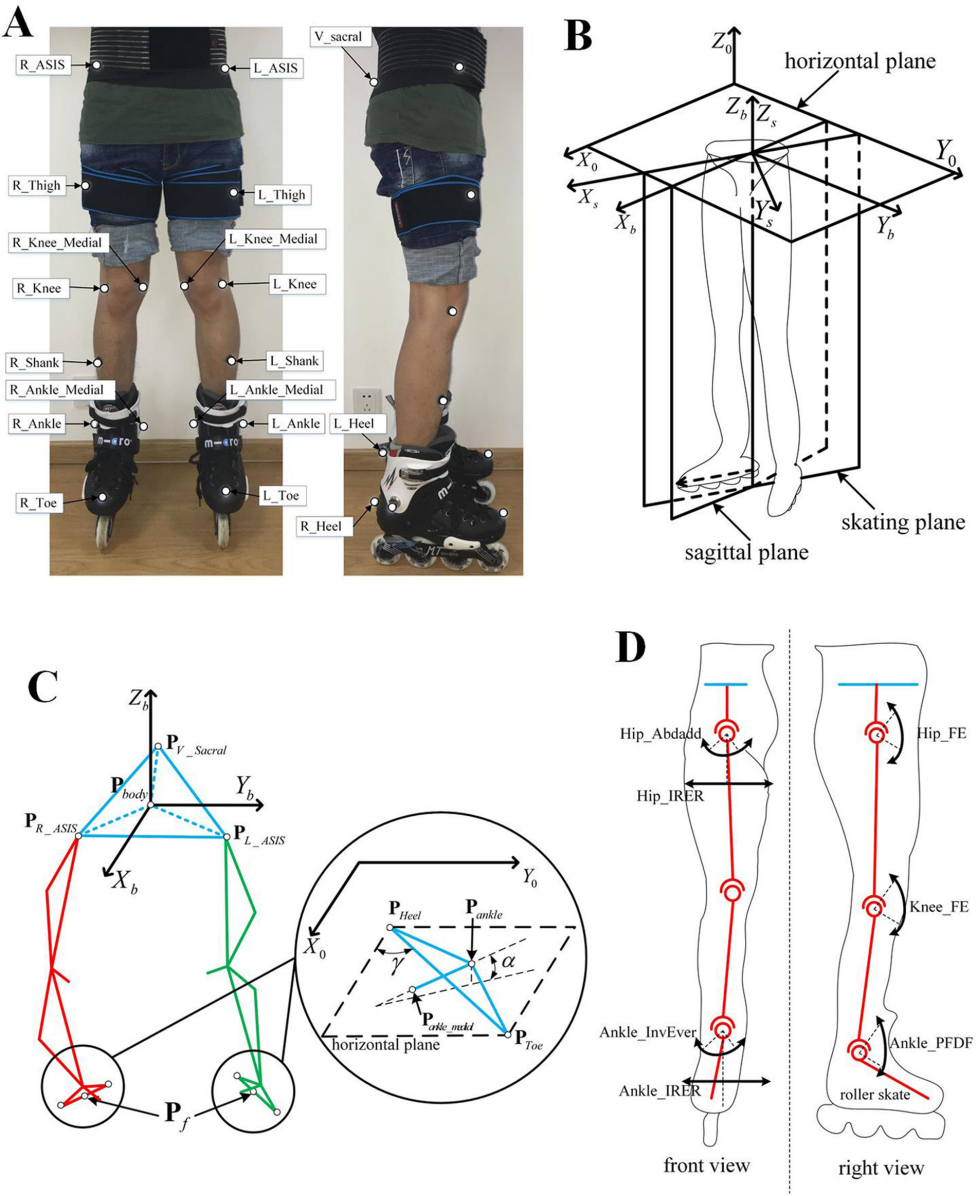
**The roller skating model.** (A) The marker set. (B) The skating plane. (C) The critical parameter. (D)The joint set.

The kinematics data were obtained by a series of human roller skating experiments using a 3D motion capture system (MotionAnalysis). The precision of MotionAnalysis system is 0.058 mm and the frequency capture of experiment is 100 Hz in this paper. In order to avoid the influence of sunlight, an indoor site with wooden floors was selected. MotionAnalysis, including eight infrared cameras (Raptor-E Specifications), was used to collect the kinematics data in a 10×3×3 m^3^ space. A new model named as the roller skating model, derived from the Helen Hayes model, was used to post points on roller skating participants (Fig. 5A). The influence of the upper body on roller skating gaits was ignored and we paid attention to the motion features of the lower body on the roller skating gait. The difference between the traditional Helen Hayes model and the roller skating model is that the traditional foot markers are installed on the roller skate. The L_Toe/R_Toe marker is installed on the toe of the roller skate. The L_Heel/R_Heel marker is installed on the heel of the roller skate. The L_Ankle, R_Ankle, L_Ankle_Medial and R_Ankle_Medial markers are installed on the ankles of the roller skate. These four markers installed on one roller skate can describe the posture of passive wheels because the roller skate is regarded as a rigid body. The motion characteristics of the body can be expressed according to these three markers: R_ASIS, L_ASIS and V_Sacral. The motion between a shank and a roller skate is expressed as an ankle motion in the roller skating model.

### Data analysis

Suppose participants are symmetrical in the sagittal plane and the initial supporting leg is the left leg. The ground and body coordination systems are shown in Fig. 5B. In the ground coordination system, *Z*_0_ is perpendicular to the ground and opposite to the direction of gravity while *X*_0_ and *Y*_0_ are set on the ground. The body coordination system whose *Z*_*b*_ parallels *Z*_0_ is built on the body. The roll angle and spin angle are expressed as the angle of rotating the x and z axis from the body coordination system to the ground coordination system, respectively.

### The skating plane

In human walking, the traditional method of kinematics analysis usually focuses on the sagittal plane. Different from human walking, the motion characteristics of roller skating are difficult to describe in the sagittal plane. The motion characteristics of roller skating are analyzed in a 3D space and skating plane. The skating plane shown in Fig. 5B is defined as a plane that is perpendicular to the horizontal plane and parallel to the direction of the passive wheel on the supporting leg. The skating coordination system based on the skating plane is shown in Fig. 5B. The skating plane is the same as the sagittal plane when the passive-wheel direction of the supporting leg is parallel to the sagittal plane. In the skating coordination system, we paid attention to the relationship between the roller skate of the supporting leg and the center of body. The center of body and the position of the supporting skate is mapped in the skating coordination system by a rotation transformation. The critical parameter of the linear and turning gait is shown in Fig. 5C.

### The critical parameter of the linear gait

The linear direction of human roller skating locomotion is parallel to the *X*_0_ axis. The linear gait experimental procedure involved the participants with an initial speed, skating along a line between two points (20 times/participant). The participants tried to maintain a stable speed in one experiment and the roller skating speed adapts from slow to fast in further repeated experiments.

Normalization processing is utilized to deal with the time and speed of human roller skating in order to eliminate the influence of repeatability error. The direction of passive wheels, position of roller skates and position of the center of body are the focus of human roller skating. The on-line of the toe (L_Toe or R_Toe) marker and the heel (L_Heel or R_Heel) marker is parallel to the direction of the passive wheels. The direction of the passive wheels can be calculated as:(1)
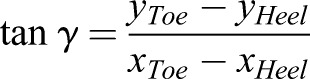
where *γ* is the angle between the *X*_0_ axis and the passive wheel, *x*_*Toe*_, *y*_*Toe*_, *x*_*Heel*_ and *y*_*Heel*_ represent the values of the toe marker and heal marker positions along the X and Y axis in the ground coordination system, respectively. The position of the roller skate can be expressed as:(2)
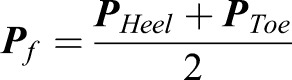

where ***P**_f_* is the position of the roller skate, ***P**_Toe_* and ***P**_Heel_* represent the position of toe (L_Toe or R_Toe) marker and heel (L_Heel or R_Heel) marker, respectively. The center of the body can be calculated as:(3)
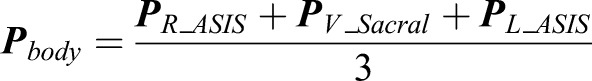

where ***P**_body_* is the position of the center of body, ***P**_R_ASIS_* ***P**_V_Sacral_* and ***P**_L_ASIS_* represent the position of R_ASIS, V_Scral and L_ASIS markers in the ground coordination system, respectively.

### The critical parameter of the turning gait

We selected left as the direction of the turning gait for consistency. The analysis method of the turning gait without swing leg is different from the linear gait. The turning gait experimental procedure involved the participants with an initial speed, skating along an arc between two points (20 times/participant). The participants tried to turn between two specific points with the same speed and turning radius.

The roll angle of the passive wheel, spin angular velocity of the passive wheel and turning radius of the passive wheel can be used to describe the motion characteristics of the turning gait. The ligature of ankle (L_Ankle or R_Ankle) marker and Ankle_Medial (L_Ankle_Medial or R_Ankle_Medial) marker is parallel to the axis of the passive wheels. The roll angle of passive wheel can be calculated as:(4)
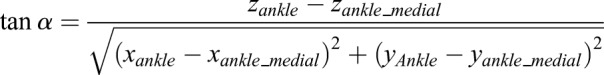
where *α* is the roll angle between the passive wheel axis and the z axis of the body, 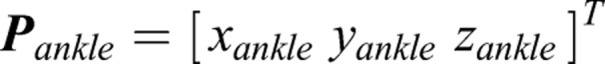 is the position of the ankle marker and 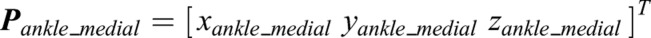 is the position of the Ankle_Medial marker. The spin angular velocity of passive wheels can be computed as the differential of γ. After obtaining the spin angular velocity of passive wheels, the turning radius of the passive wheel can be calculated as:(5)
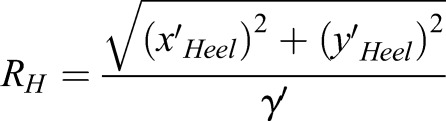
where *x*′ is the differential operation.

## Supplementary Material

Supplementary information
