# Supplementary material for: Motion characteristics of human roller skating
Source: Biol Open. 2019 Feb 20;8(4):bio037713. doi: 10.1242/bio.037713 (PMC6503993; doi:10.1242/bio.037713)
Supplement: Supplementary information [file biolopen-8-037713-s1.pdf]

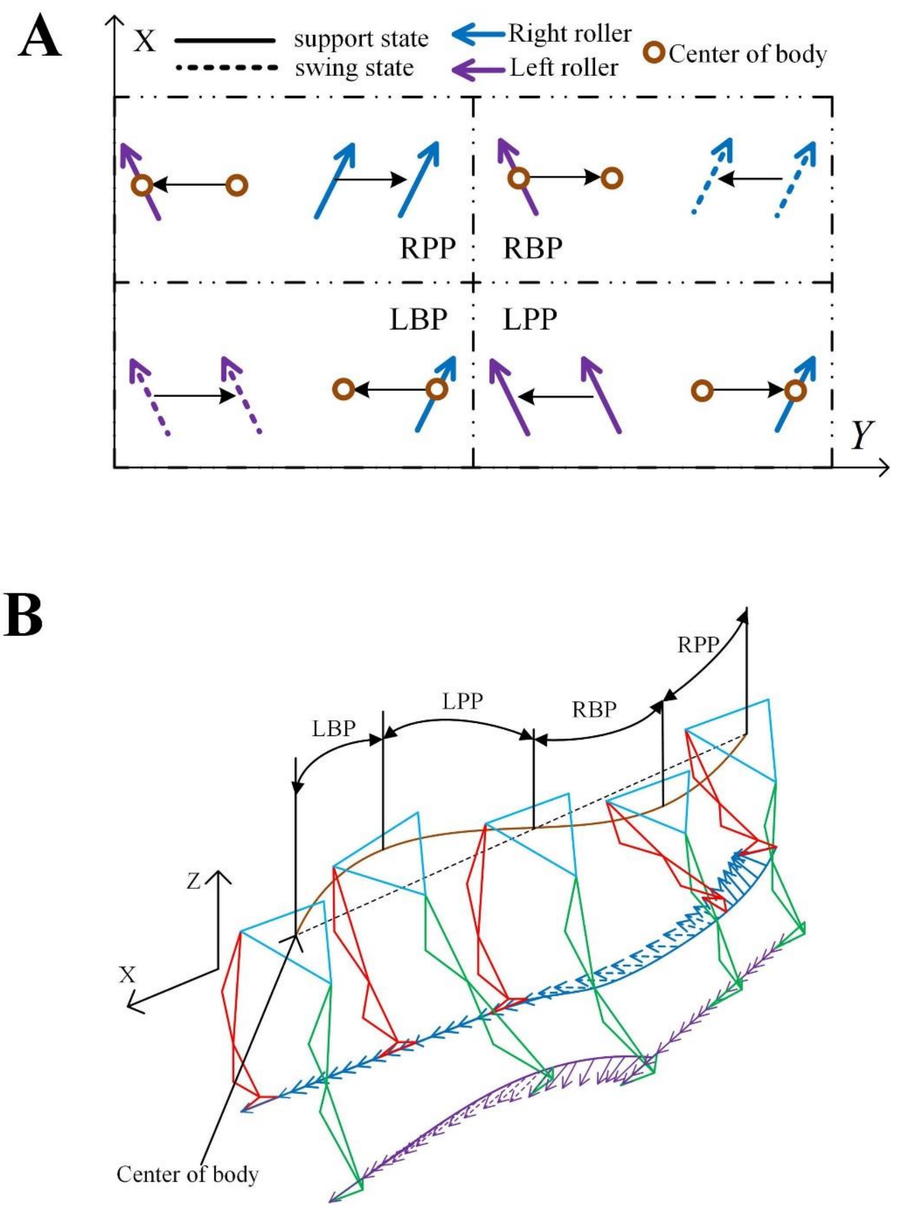

Fig. S1 The linear gait of human roller skating (A) the vertical view in body coordinate system (B) the obligue view in the ground coordinate system

Biology Open • Supplementary information

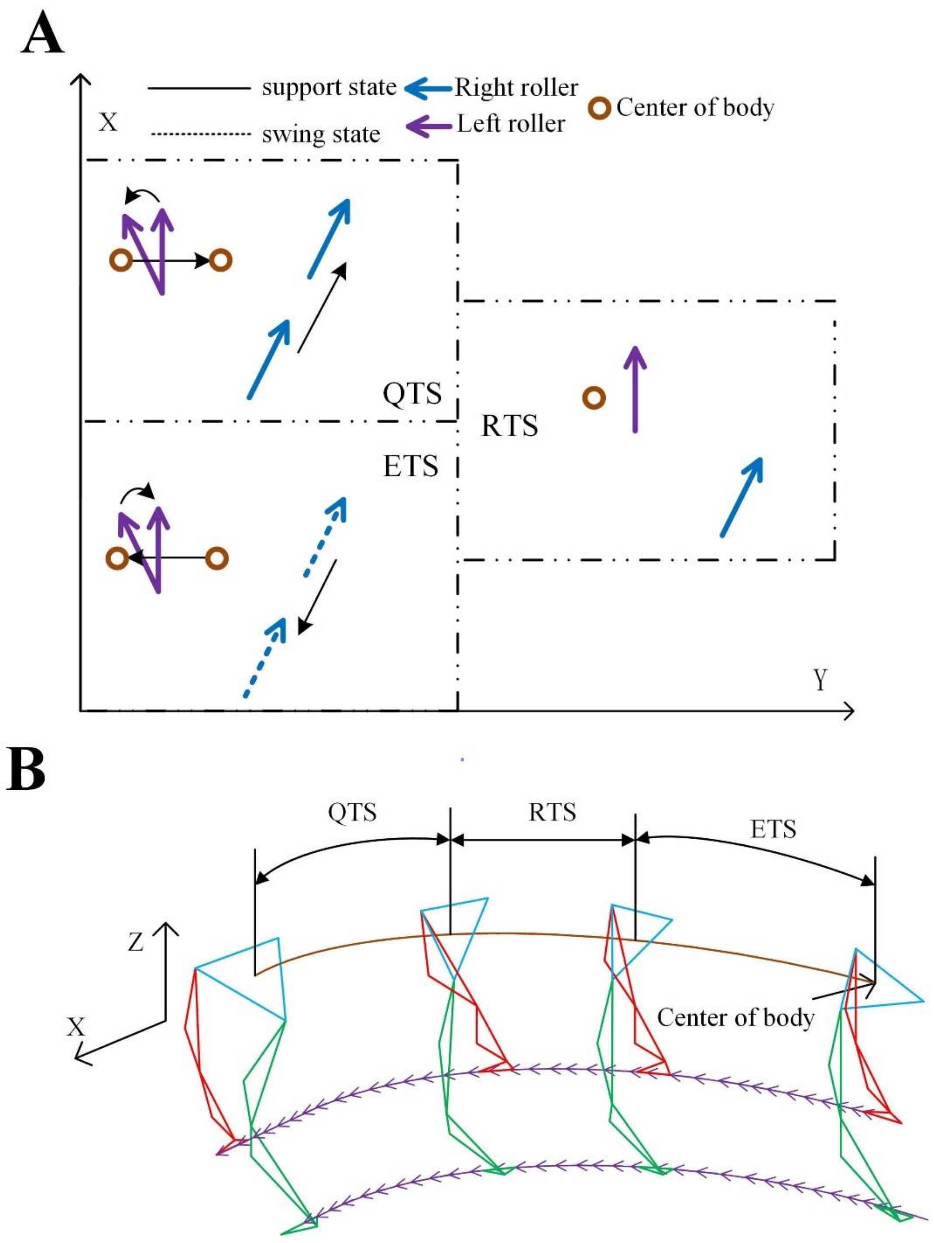

Fig. S2 The turning gait of human roller skating (A) the vertical view in body coordinate system (B) the obligue view in the ground coordinate system

Biology Open • Supplementary information

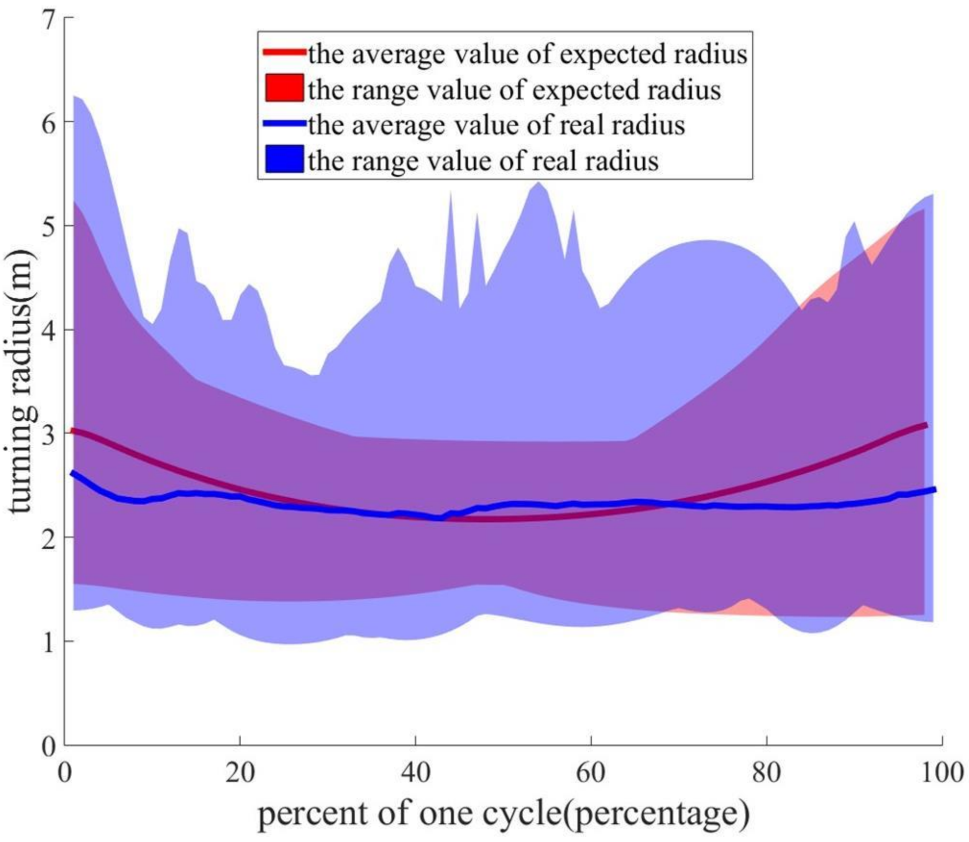

Fig. S3 The real and expected radius of body in the turning gait for all participants. The real radius of body is computed by the curvature in the ground coordinate system. The expected radius of body is calculated by the SIP model. The real radius remains about 2.5m and the expected radius changes between 3m and 2.5m.

Biology Open • Supplementary information
